# Linking the edible plant microbiome and human gut microbiome

**DOI:** 10.1080/19490976.2025.2551113

**Published:** 2025-09-04

**Authors:** Gabriele Berg, Gerardo V. Toledo, Jasper Schierstaedt, Heikki Hyöty, Wisnu Adi Wicaksono

**Affiliations:** a Institute of Environmental Biotechnology, Graz University of Technology, Graz, Austria; b Microbiome Biotechnology, Leibniz Institute for Agricultural Engineering and Bioeconomy (ATB), Potsdam, Germany; c Institute for Biochemistry and Biology, University of Potsdam, Potsdam, Germany; d Solarea Bio, Waltham, 02435 USA; e Department of Virology, Faculty of Medicine and Health Technology, Tampere University, Tampere, Finland; f Fimlab Laboratories, Pirkanmaa Hospital District, Tampere, Finland

**Keywords:** Edible plant microbiome, gut microbiome, fruits and vegetables, human health

## Abstract

The edible plant microbiome, which includes microbes in raw-eaten plants, has been recently recognized as a vehicle delivering microbes to the gut. Fruits and vegetables can carry thousands to billions of microorganisms with diverse genetic capacities on each serving. Since the ‘edible plant microbiome’ concept was introduced in 2014, notable progress has been made in understanding its microbial diversity, factors influencing it, functional traits and biomarkers, and its interconnection with the human gut microbiome. The discovery of the link between microbes in plants consumed raw and the gut microbiome establishes a possible continuum from farm to fork and health.

## Introduction

Diet is considered one of the most important factors for human health. Approximately 16 million disability-adjusted life years and 1.7 million deaths worldwide are attributable to low fruit and vegetable consumption.^1^ Following WHO suggestions, the EAT-Lancet Commission supports a plant-based diet.^1^ Adequate consumption of fruit and vegetables (five total servings/400 grams per day) reduces the risk of cardiovascular diseases, stomach cancer, and colorectal cancer. Moreover, there is growing evidence from nutritional epidemiological studies about the detailed health benefits i.e., reduced levels of systolic blood pressure, C-reactive protein, plasma glucose, and BMI, along with an increase in HDL cholesterol levels, associated with greater consumption of live microbes in the diet including those that associated to fruits and vegetables.^2^ A plant-based diet can lower greenhouse gas emissions in contrast to a meat-based diet (reviewed in^3^). Therefore, a plant-based diet is not only beneficial for human health but also planetary health.

Diet is also one crucial factor of exposure and therefore integrated into the exposome concept defined by Wild.^4^ Although diet and plant (food) microbiota are listed as exposure factors, they are not yet connected and considered in exposome studies. Nonetheless, plant microbiota has been intensively studied, especially their role in plant health, growth and resilience. The plant serves not only as a natural reservoir for plant-beneficial bacteria but also as a potential habitat for food-borne as well as opportunistic human pathogens.^5,6^ Foodborne pathogens from fruits and vegetables, which pose a considerable risk to both the agricultural sector and public health, are under research and global monitoring.^7–9^ While the latter are not frequent nor abundant members of the microbiome, the majority of microorganisms associated with fruits and vegetables and their link to humans is not yet understood.

Diet plays a critical role in influencing the composition, function, and diversity of the gut microbiome. Different dietary patterns can significantly affect the stability, functionality, and diversity of the microbial community within the gut.^10^ Studies indicate that alterations in bacterial communities, or even temporary colonization during microbiota succession, may have lasting impacts on the host’s immunological and metabolic development.^11–13^ Early childhood represents a crucial phase characterized by a major nutritional transition from a milk-based diet to a solid food-based diet. This period is also critical for the development of an infant’s gut microbiota, marking a shift from an infant-type microbiota to a child-type microbiota.^14,15^ However, there remains a limited exploration of the dietary changes that occur when infants begin consuming solid foods, especially fruits and vegetables. Furthermore, while the positive impact of fruit consumption on gut microbiota and overall human health is gaining recognition,^16–18^ the contribution of the fruit and vegetable microbiota to gut microbiota is largely unknown.

**Figure 1.**
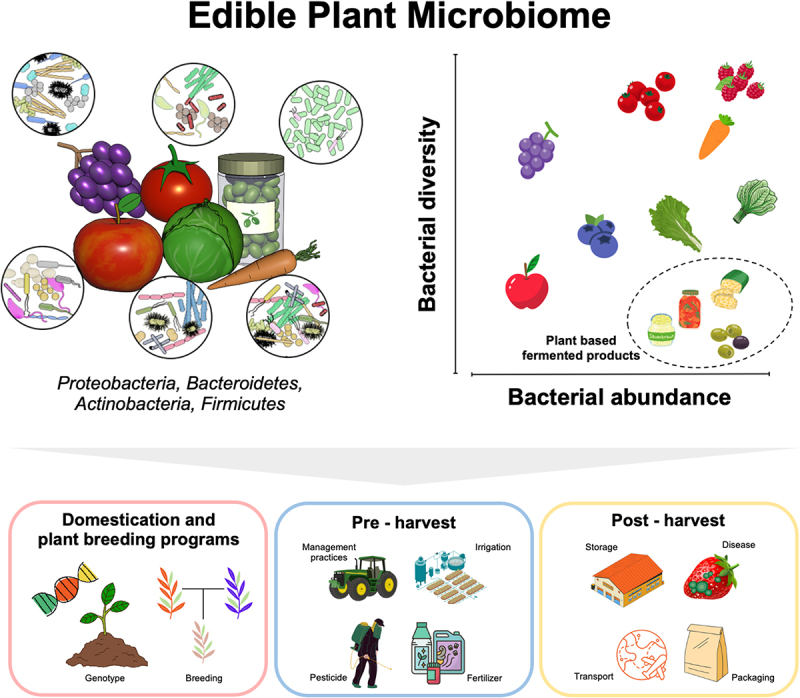
A visual representation illustrating the edible plant microbiome diversity and factors that influence the microbiome of edible plants. This figure was created using Canva (https://www.canva.com.) and BioRender (https://biorender.com). The information in the figure is derived from the reference provided in Table 1.

The concept of “edible plant microbiome” introduced in 2014^6^ has been underpinned by the results of several studies on the specific microbial diversity as well as their functional traits^19,20^ associated with fruits and vegetables. In addition to being consumed raw, fruits and vegetables can be fermented to enhance their benefits related to food preservation, safety, nutritional quality, and sensory characteristics.^21^ Fermentation is a natural process driven by microorganisms that are either inherent in the raw food source or introduced from the production environment via starter culture. Natural fermentation processes using indigenous microbes are commonly applied in the production of fermented fruits and vegetables such as fermented cereals, sauerkraut, kimchi, and soy-based products, while starter cultures are typically utilized in the manufacture of cultured dairy products, cheese, and fermented sausages.^21^ These types of foods generally exhibit a high abundance of specific bacteria, but have relatively low diversity compared to their raw materials (Figure 1). In this context, the edible plant microbiome refers to the diverse live microbial communities that are present in plants (fruits and vegetables) as well as their fermented forms. This review summarizes novel insights and attempts to elucidate the factors that influence the edible plant microbiome and their potential importance for human health. Furthermore, the edible plant microbiome is examined from the perspective of the exposome and the one health concept.

**Table 1.** Summary of research on the microbiome of fruits and vegetables, including the effects of biotic and abiotic factors on their microbiome.

Type of food product	Food product	Bacterial abundance^#^	Predominant bacterial phyla	Factor influencing	Indicator bacterial taxa*	Ref.
Fresh products	Apple	10^5^ − 10^8^	*Proteobacteria, Bacteroidetes, Actinobacteria, Firmicutes*	Domestication and breeding	*Sphingomonadales, Rhizobiales, Xanthomonadales*	^22–27^
				Growing region	*Pseudomonadales, Enterobacterales, Burkholderiales*	
				Management practices	*Pseudomonadales, Enterobacterales, Burkholderiales*	
				Post-harvest storage	*Pseudomonadales, Enterobacterales*	
				Post-harvest disease	*Pseudomonadales, Sphingomonadales, Rhizobiales*	
				Washing and waxing	*Pseudomonadales, Burkholderiales, Sphingomonadales, Rhizobiales, Actinomycetales, Bacteroidales*	
				Hot water treatment and biological control	*Pseudomonadales, Burkholderiales, Rhizobiales, Micrococcales, Cytophagales*	
				Food processing	*Pseudomonadales, Burkholderiales, Bacillales, Xanthomonadales*	
	Strawberry	10^8^ − 10^9^	*Proteobacteria, Bacteroidetes, Actinobacteria, Firmicutes*	Disease during preharvest	*Enterobacterales, Sphingomonadales, Rhizobiales*, *Rhodospirillales*	^28,29^
				Postharvest storage	*Sphingomonadales, Rhizobiales*	
				Biological control	*Pseudomonadales, Enterobacterales, Xanthomonadales*	
	Blueberry	10^5^ − 10^7^	*Proteobacteria, Actinobacteria, Firmicutes*	Growing region	*Pseudomonadales, Enterobacterales, Burkholderiales*	^24,30,31^
				Management practices	*Pseudomonadales, Enterobacterales, Burkholderiales*	
				Postharvest storage	*Burkholderiales, Sphingomonadales, Rhizobiales, Micrococcales, Flavobacteriales*	
	Grapes	10^5^	*Proteobacteria, Actinobacteria, Bacteroidetes, Firmicutes*	Management practices	*Bacillales, Actinomycetales, Clostridiales*	^32–36^
				Growing region	*Burkholderiales, Enterobacterales, Bacillales*	
	Spinach	10^6^ − 10^7^	*Proteobacteria, Firmicutes, Bacteroidetes*	Management practices	*Enterobacterales, Bacillales*	^32,37–40^
				Type of irrigation water	*Flavobacteriales, Sphingobacteriales, Rhodobacterales, Lactobacillales*	
				Postharvest handling and sanitation	*Pseudomonadales, Bacillales, Micrococcales*	
				Post-harvest storage	*Pseudomonadales, Burkholderiales, Sphingomonadales, Rhizobiales*	
	Lettuce	10^5^- 10^7^	*Proteobacteria, Firmicutes, Bacteroidetes*	Growing region	*Flavobacteriales, Geodermatophilales*	^32,40–44^
				Growing season	*Enterobacterales, Pseudomonadales, Burkholderiales*	
				Disease during preharvest	*Enterobacterales*	
				Management practices	*Pseudomonadales, Enterobacterales, Bacillales*	
	Tomato	10^4^ − 10^7^	*Proteobacteria, Firmicutes, Bacteroidetes*	Rain period	*Enterobacterales*, *Xanthomonadales*	
				Management practices	*Pseudomonadales, Rhizobiales, Sphingomonadales, Cytophagales*	^45–48^
				Fruit diseases and biological control	*Pseudomonadales, Sphingomonadales, Rhizobiales*	
	Carrot	10^8^ − 10^9^	*Proteobacteria, Bacteroidetes, Actinobacteria, Firmicutes*	Management practices	*Pseudomonadales, Bacillales, Xanthomonadales*	^49–51^
				Post-harvest storage	*Pseudomonadales, Enterobacterales, Xanthomonadales*	
	*Brassica* vegetables	10^9^ − 10^10^	*Proteobacteria, Bacteroidetes, Actinobacteria, Firmicutes*	Disease during preharvest	*Sphingomonadales, Bacillales, Streptomycetales, Pseudonocardiales*	^52–56^
				Vegetable processing system	*Pseudomonadales, Enterobacterales, Sphingomonadales, Bacillales, Flavobacteriales*	
Fermented products	Fermented olives	10^13^ − 10^14^	*Firmicutes, Proteobacteria*	Growing region	*Lactobacillales*	^57–59^
				Spoilage during fermentation	*Lactobacillales, Enterobacterales*	
	Fermented soybean	10^7^ − 10^9^	*Firmicutes, Proteobacteria*	Production region	*Lactobacillales*, *Enterobacterales*	^60,61^
				Hygienic practices	*Lactobacillales*, *Enterobacterales*	
	Kimchi	10^5^ − 10^7^	*Firmicutes, Proteobacteria*	Manufacturing season	*Lactobacillales, Pseudomonadales*	^62–64^
				Raw ingredient	*Lactobacillales, Enterobacterales, Pseudomonadales*	
	Sauerkraut	10^7^ − 10^8^	*Firmicutes, Proteobacteria*	Raw ingredient	*Lactobacillales, Pseudomonadales*	^65,66^
				Producer	*Lactobacillales*	

^#^Bacterial abundance was estimated using quantitative PCR-based methods.

*The indicator bacterial taxa were obtained from the reference material and aggregated to the bacterial order level.

## The specific microbial diversity associated with raw-eaten plants

### The edible plant microbiome community structure and composition

The edible part of plants can be colonized by thousands to billions of microorganisms per gram of plant. The edible part of plants contains a variety of microorganisms, including archaea and eukaryotes such as fungi, algae, and protists.^67^ However, this review will primarily focus on bacteria and their functional contributions as members of the edible plant microbiome. The predominant bacterial taxa found in the edible part of plants, i.e., fruits and vegetables are *Proteobacteria*, *Bacteroidetes*, *Actinobacteria*, and *Firmicutes* (Table 1). These bacterial phyla are enriched in the plant endosphere in comparison to the soil.^68^ These selective enrichments mediated by host’s immune systems and secondary metabolites within different plant compartments are known to contribute to the distinct composition differences observed between plant species and sample types.^69,70^ This pattern is also true for the edible parts of plants. For example, certain fruits and vegetables i.e., sprouts, spinach, lettuce, and tomatoes, harbored high levels of *Enterobacteriaceae* while others i.e., apples, grapes, and peaches were primarily composed of *Actinobacteria*, *Firmicutes*, *Bacteroidetes*, and *Proteobacteria*.^32^ Jarvis and colleagues^71^ also demonstrated the presence of unique microbiome profiles in food products, with cucumbers being predominantly composed of *Proteobacteria*, *Firmicutes*, and *Actinobacteria*, whereas cilantro and sprouts primarily contained *Proteobacteria* and *Firmicutes*, respectively.The identification of specific microbiomes in fruits and vegetables reveals the mutual adaptation of bacterial species and their plant hosts. A grapevine microbiome, composed of *Enterobacterales*, *Pseudomonadales*, *Bacillales*, and *Rhodospirillales* has been reported to be unaffected by environmental conditions.^72^ In arugula, *Enterobacteriaceae*, a main member of the microbiome, also exhibits a range of antibiotic resistance mechanisms that may be related to the presence of bioactive metabolites produced by the host plant.^52^ A study examining apple microbial communities from 21 locations across eight identified two bacterial genera, *Sphingomonas* and *Methylobacterium*, along with six fungal genera: *Aureobasidium*, *Cladosporium*, *Alternaria*, *Filobasidium*, *Vishniacozyma*, and *Sporobolomyces*, consistently detected across all obtained samples.^22^

### Which factors can influence the edible plant microbiome?

The fruit and vegetable microbiome is mainly influenced by the host plants, including the plant genotype. A study on the microbiome of raw *Brassica* vegetables demonstrates that genetically similar varieties harbor more similar microbial communities.^53^ A study on 11 *Malus* species, representing the domesticated apple (*M. domestica*), wild apple progenitors, and wild *Malus* species found significant connections between host phylogenetics and microbiome similarity.^73^ Interestingly, the apple microbiome is also collectively shaped by spatial variation in microbial communities including peel, stem-end, calyx-end, and mesocarp.^23,74^ These findings suggest that the microbiome of fruits and vegetables is diverse and structurally assembled.

Domestication and plant breeding programs have not only resulted in varieties with improved yields and resilience against pathogens^75^ but also influenced the edible plant microbiome. For example, apple domestication leads to higher fungal diversity and an increase in microbial population size due to an increased niche size or amount of resources in domesticated apples.^73^ The ‘Monterey’ cultivar of strawberry demonstrated higher productivity and larger fruit size, along with a higher tolerance to leaf spot and powdery mildew in comparison to the ‘Elsanta’ and ‘Darselect’ cultivars.^76^ Interestingly, the ‘Monterey’ cultivar also contains a higher number of IAA-producing bacteria and antagonists of *Xanthomonas fragariae*, the causal agent of leaf spot disease suggesting their potential role in plant growth promotion and protection.^76^ Surface properties of fruits and vegetables, such as texture, surface topography, and moisture content, can affect the attachment and colonization of microorganisms.^77,78^ The presence of waxy cuticles or natural antimicrobial compounds on fruits may affect the adherence and survival of microbial communities.^79^ This, in turn, can lead to changes in the composition of the microbial community. Changes in sugar content of various fruits due to ongoing selection and breeding practices can have varying impacts on microbial ecology by influencing microbial growth and behavior mainly enriching copiotrophic microorganisms.^80^

The edible plant microbiome is also shaped by external biotic and abiotic factors from the field to the post-harvest. The presence of biotic stresses from *Rhizoctonia* infections resulted in shifts in *Enterobacteriaceae* community structure in lettuce. Interestingly, asymptomatic *Brassica napus* roots exhibit a higher microbial diversity and abundance compared to symptomatic roots affected by clubroot disease caused by *Plasmodiophora brassicae*.^55^ This study found that bacterial taxa with plant growth promotion and biocontrol properties i.e., *Bacillaceae*, *Streptomycetaceae*, and *Sphingomonadaceae* are more abundant in asymptomatic plants. The decrease in microbial diversity and abundance of beneficial bacteria i.e., *Actinobacteria*, *Sphingomonas*, and *Methylobacterium* was also observed in strawberries infected by *Botrytis cinerea*.^28^ Interestingly, the application of biological control, such as *Metschnikowia fructicola* in strawberries^29^ and *Aureobasidium pullulans* S2^47^ in tomatoes, reduced incidence of fungal disease and increased bacterial diversity. This includes the presence of beneficial bacterial and fungal taxa. These findings indicate that a high microbial diversity is linked to healthy fruits and vegetables whereas infections of plant pathogens led to a decrease in microbial diversity and a reduction in potentially beneficial bacteria.

Various abiotic factors also influence the edible plant microbiome. In the field, natural abiotic factors such as rain period, growing region, and growing season were reported to affect the fruit and vegetable microbiome (Table 1). Several indicator taxa, including *Pseudomonadales*, *Burkholderiales*, and *Enterobacterales*, were consistently affected by abiotic factors in the field. There is a significant amount of research focused on studying the effects of human-made abiotic factors that commonly occur post-harvest i.e., washing, waxing, and hot water treatments on the composition and abundance of microbial populations found in fruit and vegetables.^26,29,81,82^ Moreover, an increase in the abundance of *Enterobacterales* in apples following storage and transportation was also observed.^25^ This shift also leads to a higher variety of antimicrobial-resistance genes (ARGs) present. The preparation and cooking of apples can also influence the edible plant microbiome belonging to the *Bacillus* genera, which possess potential probiotic properties.^27^ It is noteworthy that certain bacterial taxa that are affected by post-harvest treatments such as *Pseudomonadales*, *Sphingomonadales*, *Enterobacterales*, and *Rhizobiales* (Table 1) have also been identified in the human gut.^20^ Changes in the microbial composition of the edible plant microbiome community caused by biotic or abiotic can significantly impact the production yield and quality of fruits and vegetables.

## Evidence for continuum along the food-gut microbiome axis

### Evidence of plant-associated microbes colonizing the human gut

Early evidence of plant-associated bacteria colonization in animals and humans could be found in studies in mammals (Table 2). An increase in bacterial diversity from carnivory to omnivory to herbivory mammals was reported.^83^ As ancestral mammals were carnivorous, the authors aimed to determine whether the bacterial lineages present in herbivores were derived from those found in carnivores. The results did not support this hypothesis, indicating that mammals that rely on a plant-based diet probably acquired their gut microbiota from the environment. Some typical gut bacteria of herbivores i.e., *Lactobacillus*, *Clostridium*, *Enterobacter*, *Streptomyces*, *Rhodococcus* and *Microbacterium* are frequently identified as plant endophytes.^90^ Plant endophytes are expected to possess enzymes capable of breaking down cell walls^91^ and would be beneficial for animals to degrade plant polysaccharides in their digestive tracts. The above-mentioned evidence supports the notion that plants can serve as a source of microbial inoculum for animals. Food-borne microorganisms i.e., *Lactococcus lactis*, *Pediococcus acidilactici*, and *Streptococcus thermophilus* also survived in the human digestive system following the consumption of plant and dairy products.^84^ Furthermore, research indicated that dietary habits of rural Bedouins which consist of a variety of vegetables, fruits, and fermented products led to a higher bacterial diversity in their gut as compared to urban Saudis, who typically have a limited intake of vegetables and fruit.^85^

**Table 2.** Examples of studies on the transmission of the edible plant microbiome to the human gut.

Study	Sample Size	Method	Result
Ley et al.^83^	106 mammal fecal samples	Clone library − 16S rRNA gene amplicon sequencing	Bacterial diversity increases from carnivory to omnivory to herbivory. Plant-based mammals likely acquire gut microbiota from environments.
David et al.^84^	9 individuals subjected two diet arms − 236 fecal samples 56 food samples	16S rRNA- and ITS genes amplicon sequencing and RNA-Sequencing	Short-term consumption of animal and plant-based diets influences the gut microbial communities. Foodborne microorganisms can be detected in the human digestive system.
Angelakis et al.^85^	28 human fecal samples - urban and rural lifestyles 34 baboon fecal samples 6 fermented food samples	16S rRNA gene amplicon sequencing	Consumption of vegetables and fruits increases bacterial diversity. Fermented food-associated bacteria were more abundant in Bedouins than in urban Saudis. Bedouins’ gut microbiome closely resembles baboons’ gut microbiome, possibly due to dietary overlap.
Pasolli et al.^86^	303 food samples 9,445 human fecal samples	Shotgun metagenome	The discovery of closely related LAB strains in food and gut environments suggests that fermented foods may be a potential source of LAB for the gut microbiome.
Wicaksono et al.^20^	156 fruit and vegetable samples 2,426 human fecal samples	Shotgun metagenome	Consistent presence of fruit and vegetable bacteria, but at low abundance. Factors influencing diversity include host age, vegetable consumption frequency, and plant diversity.
Mantegazza et al.^87^	Fecal samples from 23 individuals	in vitro simulation of the gastrointestinal transit, and 16S rRNA gene amplicon sequencing	Bacteria associated with raw vegetables can survive gastrointestinal transit.
Carlino et al.^88^	2,533 food samples (192 plant-based food samples) 19,833 fecal and oral human samples	Shotgun metagenome	Food microbes contribute to approximately 3% of the adult gut microbiome on average. Strain-level analysis has identified potential occurrences of transmission from food to the gut and subsequent intestinal colonization.
Fackelmann et al.^89^	21,561 individuals - fecal samples from 656 vegans, 1,088 vegetarians and 19,817 omnivores	Shotgun metagenome	Typically plant and soil microbes are detected in the human gut. Plant-associated microbial pathways are more prominent in vegetarian and vegan diets.

### Tracing the microbes along the food-gut axis

One important question that should be considered is the extent to which environmental microbiota contributes to the gut microbiome diversity. The mechanisms underlying the transmission of the human microbiome are not fully understood. However, certain bacterial genera are suggested to be acquired from the environment including fresh produce.^86^ For instance, fresh fruits and vegetables are at risk of contamination by enteric bacterial pathogens such as *Salmonella enterica*, *Escherichia coli*, and *Listeria monocytogenes*.^92^ These pathogens can proliferate on fruits and vegetables, colonize the human gut, and cause diseases upon consumption. It is important to recognize that these pathogens are not typical members of the fruit and vegetable microbiome. In the past year, numerous studies have indicated the transmission of microbiomes associated with fruits and vegetables through various methods, including shotgun metagenomics and *in vitro* gut models (Table 2). Results from *in vitro* gastrointestinal digestion simulations also indicated that bacteria associated with rockets, particularly lactic acid bacteria (LAB), can survive transit.^87,93^ Moreover, fruit- and vegetable-associated bacteria are consistently detected in the human gut, although at a low abundance ranging from 0.003% to 3.1%.^20^ A recent study analyzing over 20,000 human metagenomes revealed that bacteria associated with food, including those derived from fruits and vegetables, accounted for an average of 3% of the adult gut microbiome.^88^ The above-mentioned evidence indicates that microbes derived from fruits and vegetables can be transmitted, colonize the human gut, and contribute to the diversity of the gut microbiome.

**Figure 2.**
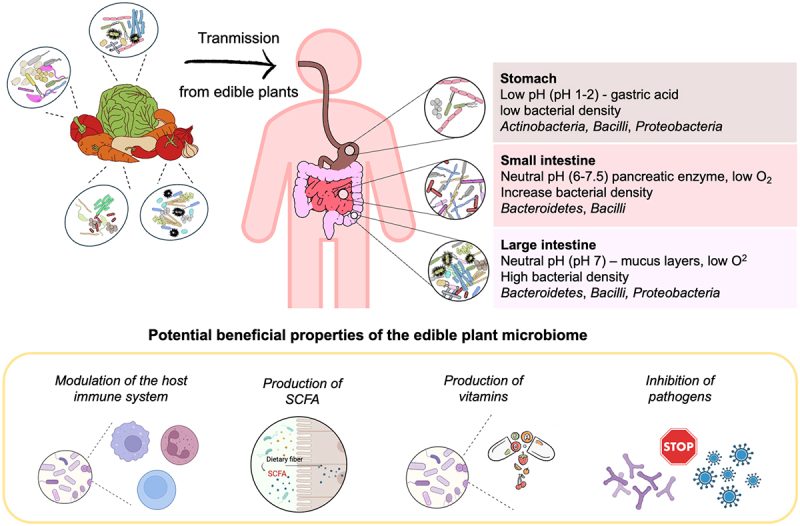
Microbial transmission from plants to the human gastrointestinal system and its potential implications for human health. This figure was created using Canva (https://www.canva.com.) and BioRender (https://biorender.com).

Another fundamental question is which bacterial taxa are likely to be transmitted from fruits and vegetables consumed raw to the gut. There is minimal taxonomic overlap observed between fruits, vegetables and the human gut comprising members of the *Proteobacteria*, *Bacilli*, *Actinobacteria*, and *Bacteroidetes* groups^90,94^ (Figure 2). To be transmitted and establish a (transient) colonization in the gut, bacteria must adapt to various gut environments. For example, the low pH of gastric fluid in stomach and the oxygen gradient present in the small and large intestine serve as significant ecological filters.^95^ Among these phyla, lactobacilli as well as several bacterial genera such as *Acinetobacter*, *Bacillus*, *Enterococcus, Leuconostoc*, *Weissella*, *Enterobacter*, *Pantoea*, *Pseudomonas*, *Streptomyces* and *Xanthomonas* were typically recovered from both fresh produces and the human gut.^20,22,96,97^ Facultative anaerobic bacteria, such as *Pantoea, Pseudomonas, and Acinetobacter* have been observed to occasionally colonize the gastrointestinal tract.^20^ These bacteria have evolved a wide range of strategies to tolerate varying levels of oxygen and anaerobic conditions, allowing them to adapt effectively to cellular hypoxia.^98^ *Bacillus* and *Streptomyces*, which are also common members of the plant microbiome, can produce metabolically dormant and highly resistant spores. These spores can become activated and may potentially facilitate colonization within the human gastrointestinal system.^99,100^ *Bacteroidetes* species, commonly present in fresh produce and fermented foods, are specialized in the breakdown of high-molecular-weight organic compounds. They possess a diverse array of carbohydrate-active enzymes that target a broad spectrum of substrates from plant, algal, and animal sources,^101^ that could support their colonization in the human gastrointestinal tract. Additionally, bacterial genera *Lactiplantibacillus* and *Lactococcus*, which is frequently found in various fresh produce and fermented food products, is also commonly detected in the human gut.^20,88^ These taxa are recognized for their capacity to produce EPS, which contributes to the protection of microbial strains against acid and bile stress within the gastrointestinal tract.^102^ Additionally, the selective adhesion of these taxa to epithelial cells appears to be a key mechanism facilitating their stable association with host organisms.^103,104^ Extending the distribution range of *Lactobacillus, Bacillus*, and *Enterococcus* into human nutrition, these genera are frequently used in probiotic products.^105^ Furthermore, the genus *Bifidobacterium*, which belongs to the *Actinobacteria* phylum, is a common inhabitant of the gastrointestinal tract. Several species, such as *B*. *longum*, *B*. *breve*, and *B*. *infantis*, are recognized for their probiotic properties (reviewed in^106^). Notably, this genus is also present in various food products, suggesting a potential pathway for the transmission of these taxa to the human gut.^88^ This suggests that fruits and vegetables may serve as a source of beneficial microbes with health potential and partially could explain why these dietary items are associated with healthy outcomes.

*Lactobacillus* —the genus previously classified under this name has recently been redefined to include a total of 25 distinct genera^107^—is the most extensively researched group within the lactic acid bacteria (LAB).This group serves as a model for understanding the interconnection between plants and humans. This genus can be classified into three groups: free-living, host-adapted, and nomadic.^104^ Species with nomadic lifestyles such as *L. plantarum*, *L*. *rhamnosus*, and *L. casei*, exhibit large genomes that are associated with enhanced metabolic adaptability. Their adaptability and resilience in various ecological and metabolic conditions might allow them to migrate across different environments such as plant tissues and the digestive systems of mammals.^97^ Interestingly, comparative genome analysis of LAB, including *Lactobacillus* strains revealed that closely related strains are present in plants and the human gut.^86^ This result suggests a possible bacterial transmission between these two environments and that the same species can be adapted to live in both plant and animal host as part of its life cycle. Interestingly, the detection of plant-associated bacteria belonging to genera such as *Lentilactobacillus*, *Lactobacillus*, and *Lactococcus* has shown a gradual increase in abundance during the early stages of life,^20^ suggesting that these taxa potentially persist over time in the human gut and might play a role in the host’s health. As plant-associated LAB exhibit phenotypic and genotypic diversity with distinct traits,^97^ fruits and vegetables have the potential to emerge as a novel source of probiotics.

## Exploring putative beneficial properties of the edible plant microbiome

Lactic acid bacteria species originated from food and vegetables (Table 3) have been the subject of extensive research concerning their beneficial properties for human health including the modulation of the host immune system and the production of beneficial metabolites. In addition to lactic acid bacteria, non-lactic acid bacteria isolated from fruits and vegetables, including those found in fermented fruits and vegetables, have also shown potential beneficial effects (Table 3). However, research on their importance remains considerably less developed compared to that of the lactic acid bacteria group. The proposed effects of the non-lactic acid bacteria were primarily demonstrated *in vitro*, highlighting the necessity for *vivo* studies to explore their beneficial impacts.

**Table 3.** Summary of potential beneficial properties of the edible plant microbiome.

Food product	Bacterial strains	Putative health benefit	Study type	References
Carrot	*Lactobacillus plantarum*	Inhibition of bacterial pathogens Stimulation of cytokines Production of organic acids	In vitro	^108^
Tomato	*Lactobacillus plantarum*			
Pineapple	*Lactobacillus plantarum*			
Papaya	*Enterobacter tabaci*	Inhibition of bacterial pathogens	In vitro	^109^
Dragon fruit	*Pantoea stewartii*			
Sugar apple	*Microbacterium*			
	*Microbacterium*			
Apple	*Bacillus* spp.	Inhibition of bacterial pathogens	In vitro	^27^
	*Pseudomonas* spp.	Production of biosurfactants		
Bitter apple	*Nocardiopsis alba*	Inhibition of bacterial pathogens	In vitro	^110^
	*Streptomyces mediolani*			
Marula	*Lactobacillus plantarum*	Bacteriocin-like substances	In vitro	^111^
Bell pepper	*Enterococcus faecium*	Antimicrobial activity	In vitro	^112^
Cucumber	*Leuconostoc mesenteroides*	Proteolytic activity	In vitro	^112^
		Exopolysaccharide production		
Red apple	*Enterococcus mundtii*	Proteolytic activity	In vitro	^112^
Tangerine	*Leuconostoc mesenteroides*	Amylase activity	In vitro	^112^
		Exopolysaccharide production		
Mung bean sprouts	*Lactococcus lactis*	Carbohydrate metabolisms	In silico	^113^
Radish, Lettuce, Apple	*Pseudomonas*	Production of vitamin B_12_	In vitro	^20^
	*Nocardioides*			
	*Aeromicrobium*			
	*Microbacterium*	Production of vitamin K	In vitro	^20^
	*Arthrobacter*			
	*Nocardioides*			
A mix of fresh fruits, vegetables, and fermented foods	*Lactiplantibacillus plantarum*	Production of short-chain fatty acids	In vitro and In vivo	^114^
	*Levilactobacillus brevis*	Production of vitamin K		
	*Leuconostoc mesenteroides*	Modulation of the gut microbiome		
	*Pseudomonas fluorescens*			
Medicinal plants *Mirabilis jalapa* and *Clerodendrum colebrookianum*	*Streptomyces* sp.	Inhibition of bacterial pathogens	In vitro	^115^
	*Microbacterium* sp.			
	*Leifsonia xyli*			
	*Brevibacterium* sp.			
Kimchi	*Lactobacillus brevis*	Antioxidant activity Enhances immune function	In vitro	^116^
Fermented baobab seed	*Bacillus subtilis*	Bacteriocin-like substances	In vitro	^117^
Fermented mustard	*Lactobacillus plantarum*	Production of vitamin B_12_	In vitro	^118^
Cabbage kimchi	*Latilactobacillus sakei*	Production of antioxidants	In vitro	^119^
	*Latilactobacillus graminis*	Antibacterial Properties		
	*Bacillus proteolyticus*			
Chinese fermented cabbage	*Lactobacillus plantarum*	Bacteriocin-like substances	In vitro	^120^
Fermented cucumber	*Lactococcus garvieae*	Bacteriocin-like substances	In vitro	^121^
Pickled Chinese celery	*Enterococcus faecium*	Bacteriocin-like substances	In vitro	^122^
Fermented bamboo	*Enterococcus faecalis*	Bacteriocin-like substances	In vitro	^123^
Fermented black beans	*Enterococcus faecium*	Bacteriocin-like substances	In vitro	^124^
Fermented soybean	*Azotobacter* sp.	Production of vitamin B_12_	In silico	^60^
	*Acetobacter* sp.			
Fermented soybean	*Leuconostoc* sp.	Production of vitamin K	In silico	
	*Weissella* sp.			
	*Enterococcus* sp.			
	*Enterobacter* sp.			

### Modulation of the host immune system

Fruit and vegetable-associated bacteria that enter the gastrointestinal system can interact with members of the gut microbiome and the host immune system. This is known for probiotics such as *Lactobacillus* and *Lactococcus*^125^ similar to those present in fruits and vegetables. For example, a study conducted on newborns identified as high risk for asthma has shown that the consumption of *Lactobacillus rhamnosus* GG can lead to notable alterations in the composition and functionality of the early gut microbiota.^126^ This treatment also induced the development of T-regulatory cells. Recently, a novel probiotic strain of *Levilactobacillus brevis* was isolated from radish kimchi and demonstrated immune-enhancing effects by increasing cytokine production of inducible nitric oxide synthase (iNOS) and TNF-α.^116^ *Paenibacillus polymyxa*, a known plant growth-promoting rhizobacterium, has been used as a probiotic in animal applications. The administration of this probiotic enhanced the activity of iNOS that modulates proinflammatory activation in the liver and jejunal mucosa and increased the production of proinflammatory cytokines in broilers subjected to probiotic treatment.^127^ In another study, the extracellular polymeric substances derived from *P. polymyxa* strain CCM 1465 have been shown to influence the production of the proinflammatory cytokine TNF-α and activate various immune cells.^128^ Furthermore, *Enterobacteriaceae*, which is one of the prevalent groups found in fruits and vegetables, encompasses several species known to be opportunistic pathogens. Notably, the endotoxins produced by these bacteria have been identified as both protective and enhancing factors in asthma, contributing to allergy protection and immunomodulation.^129^ Furthermore, lipopolysaccharide derived from *E. coli* has been shown to induce endotoxin tolerance *in vivo* in NOD mice and may contribute to a reduced incidence of diabetes in mouse model.^130^ Therefore, exposure to nonpathogenic plant-associated bacteria may affect the development of allergies^32^ and raw produce consumption may introduce new commensal bacteria into the human gastrointestinal system, potentially affecting host immune systems and disease development.

### Production of beneficial metabolites

Bacteria present in fruits and vegetables have the potential to produce beneficial metabolites. Bacterial strains isolated from cereals, fruits, and vegetables i.e., *L*. *reuteri* and *L. plantarum* were identified as vitamin B_12_ producers.^118,131^ A recent study revealed that *cobC*, a gene responsible for synthesizing vitamin B_12_ was detected in bacterial genomes originating from fruit and vegetable samples.^20^ Bacterial genomes belonging to *Actinomycetales*, *Pseudomonadales*, and *Propionibacteriales* also contained genes associated with the biosynthesis of vitamin K_2_ and the production of short chain fatty acids (SCFAs).^20^ A mouse model for postmenopausal osteoporosis with probiotic strains coding for vitamin K_2_ biosynthesis and producing SCFA synergistically showed bone-protecting effects in the OVX mice.^114^ *Azotobacter* and *Acetobacter* strains derived from fermented soybean (tempeh) possess genes responsible for producing vitamin B_12_.^60^ Additionally, *Enterobacter* (*Enterobacteriaceae*), which may primarily originate from soybeans, also contains genes linked to the production of vitamin K.

Quorum sensing (QS) molecules play a significant role in the interactions within the diverse bacterial community. These molecules are produced by both human and plant-associated bacteria, such as *N*-acyl homoserine lactone, which has been shown to influence gut immunity as well as the immune systems of plants.^132–134^ These signaling molecules can lead to cooperative alterations in bacterial gene expression, such as the expression of virulence factors, as well as changes in bacterial behaviors, including biofilm formation.^134^ Interestingly, in some strains of *L. plantarum* derived from fruits and vegetables, quorum sensing is involved in the production of bacteriocins with antimicrobial activities.^135^ Furthermore, QS appears to play a significant role in bacterial colonization. For example, a high level of QS ie., Autoinducer 2 has been associated with increased abundance of lactic acid bacteria on the surfaces of olives,^136^ supporting the notion that a strong biofilm-forming capacity can enhance the survival and establishment of potential probiotic strains within fermented food microbiomes.^136^ Bacteria with deficiencies in QS are less effective at colonizing the gut, a trend observed in commensal, probiotic, and pathogenic strains^134^ indicating their role in bacterial colonization.

Other secondary metabolites, such as secondary bile acids and aromatic amino acid derivatives produced by plant-associated bacteria, may also influence human gut health. Microbial metabolism of bile acids plays a vital role in maintaining intestinal homeostasis and modulating the development of human diseases, such as metabolic disorders (reviewed in^137^). A systematic analysis of secondary bile acid production genes in the global microbiome revealed that bile salt hydrolase genes involved in bile acid metabolism are predominantly present in *Firmicutes*, including genera such as *Lactiplantibacillus* and *Enterococcus*—both of which are associated with plants and humans.^138,139^ Furthermore, the distribution of secondary bile acid production genes was observed across various environments globally, including in mammalian hosts like humans, pigs, and mice, as well as in diverse environmental settings. Interestingly, a study indicated that *L. plantarum*, originating from olives and exhibiting bile acid metabolism capabilities, may influence host metabolic processes by modulating the bile acid pool and associated receptor signaling pathways.^140^ Many plant-associated bacteria commonly referred to as plant growth-promoting bacteria (PGPB) have the ability to synthesize indole-3-acetic acid (IAA), often utilizing tryptophan as a precursor.^141^ The IAA produced by plant-growth-promoting bacteria can enhance bacterial stress tolerance and facilitate plant growth. Interestingly, gut microbiota-derived tryptophan metabolites, such as IAA, can also influence intestinal immune responses and host physiology. These metabolites act as ligands for the aryl hydrocarbon receptor (AHR), a transcription factor in immune cells, which can modulate innate and adaptive immune responses [reviewed in ^142, 143^. These findings establish a connection between edible plant microbiome and production of secondary bile acids, as well as aromatic amino acid derivatives, which may have implications for overall gut and human health.

### Carbohydrate metabolism and plant fiber degradation

Dietary fiber consists of plant-based carbohydrate polymers that cannot be broken down by the digestive enzymes produced by the human body. Plant-associated microbes may possess fiber-degrading capabilities to break down plant polysaccharides in the human digestive system.^90^ The fermentation of dietary fiber by microbiota in the human gut has been linked to various health benefits, such as lower cholesterol levels and improved glucose control.^144^ The intestinal epithelium is protected by mucus, a matrix that is primarily comprised of large glycoproteins known as mucins. It serves as a substrate for beneficial bacteria, which play a crucial role in regulating the host immune system and are important for promoting bacterial adhesion to the gut.^145^ Interestingly, commensal bacteria have adapted to colonize the colonic mucus due to the presence of enzymes specialized in glycan utilization.^146^ The first evidence of in vitro *N-glycan* degradation was demonstrated by a plant pathogen *X*. *campestris* pv. Campestris.^147^ *Nitrospirillum amazonense* isolated from sugarcane, also contains a gene cluster responsible for N-glycan degradation.^148^ Plant-associated lactic acid bacteria also harbor a high number of genes related to carbohydrate metabolism.^97^ For instance, *Lc. lactis*, isolated from mung bean sprouts, possesses gene sets that facilitate the degradation of complex plant polymers, including xylan, arabinan, glucans, and fructans.^113^ Interestingly, carbohydrate-active enzyme profiles of vegetable and human isolates exhibited similarities.^149^ It would be intriguing to explore whether the enzyme repertoire of the edible plant microbiome could enhance its ability to transiently colonize and regulate the host immune system.

### Production of bacteriocins

Numerous bacteria can produce antimicrobial peptides called bacteriocins including plant-associated bacteria. Bacteriocin-producing bacteria i.e., *Lc*. *lactis*, *E*. *durans*, *L*. *plantarum*, *E*. *faecium*, *W*. *hellenica*, *L*. *garvieae*, *E*. *faecalis* and *B. subtilis* have previously been isolated from both raw and fermented fruits and vegetables (reviewed in^150^). *Bacillus subtilis*, isolated from fermented baobab seeds, exhibited inhibitory activity against *B. cereus*.^117^ Genes involved in the production of bacteriocins were also detected in plant-based foods-associated bacteria^151^ as well as from fermented food samples i.e., pickled vegetables, radish, beet kvass, and sauerkraut according to shotgun metagenomic data.^152^ Consequently, the production of bacteriocins can improve the quality and safety of foods by eliminating harmful bacteria. Bacteriocins may facilitate the colonization of the producing organism in the human gut and offer protective advantages to the host against pathogens.^153^ Bacteriocins have also been identified in plant-associated bacteria beyond the lactic acid bacterial group. For instance, *Streptomyces pluripotens* that were isolated from mangrove produces a broad-spectrum bacteriocin effective against the methicillin-resistant *Staphylococcus aureus* (MRSA) strain, *Salmonella typhi*, and *Aeromonas hydrophila*.^154^ Furthermore, the genome of *S. wadayamensis*, isolated from *Citrus reticulata*, contains a biosynthetic gene cluster associated with the production of bacteriocin.^155^ Therefore, certain edible plant microbiomes that produce bacteriocins can potentially inhibit the growth of harmful microorganisms by releasing inhibitory substances and through competitive exclusion mechanisms in the human gut.

## Integrating the edible plant microbiome into human and planetary health as well as in the exposome concept

### The edible plant microbiome plays a key role in connecting environmental microbiomes with the human microbiome

The plant microbiome has been thoroughly researched, yet it is important to acknowledge its interconnectedness with other microbiomes. One example is the potential transfer of antimicrobial resistance genes from plants via food consumption to the human gut. Antimicrobial resistance is frequently observed among bacterial species in complex ecosystems including the edible plant microbiome.^25,46,52,156,157^ Antimicrobial resistance genes identified in fresh produces, carry considerable implications for food safety and public health. Given that these fresh products are often consumed in their raw state, these interfaces can play a role in the transmission microbes and their genes i.e., ARGs^158^ to the human gut. Our understanding of microbiome transfer and connectivity is still developing and has predominantly focused on the negative aspects of microbial transmission, particularly foodborne pathogens. In contrast, the potential benefits and positive aspects of microbial transmission have not received equal consideration, despite their significant impact and importance being discussed recently.^159^ For example, a recent study demonstrated that plant-based diets can lead to the enrichment of more pronounced plant-associated microbial pathways, including d-galactose degradation and chorismate biosynthesis^89^ which are crucial for the production of essential metabolites. It is intriguing to consider that exposure to plant microbiomes through a plant-based diet may facilitate the transmission of beneficial microbes with beneficial metabolic pathways to the gut. The edible plant microbiome plays a crucial role in linking environmental microbiomes to the human microbiome.

### The edible plant microbiome and the human microbiome within the frameworks of one health and planetary health

**Figure 3.**
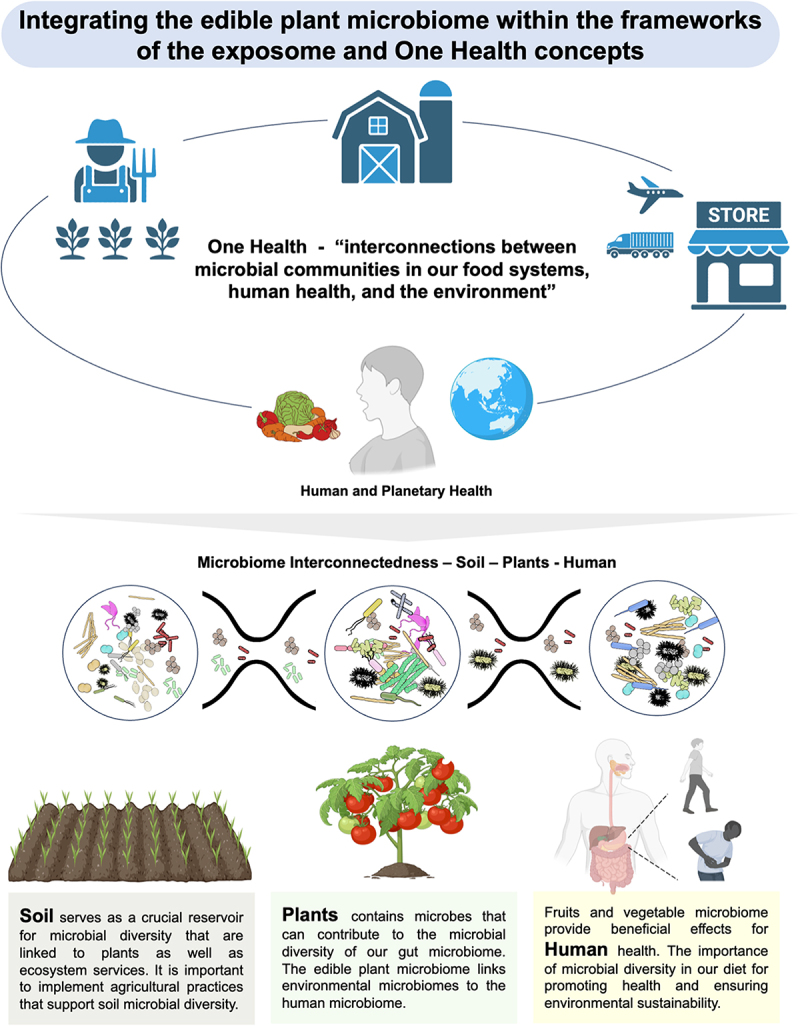
The edible plant microbiome and one health. The edible plant microbiome serves as an important connection between environmental microbiomes and the human microbiome. This figure was created using Canva (https://www.canva.com.) and BioRender (https://biorender.com).

World Health Organization has introduced the One Health concept, which acknowledges the interconnectedness and interdependence of human, animal, plant health, and the overall environment. Sustainable agricultural practices that promote microbial diversity in soil and plants contribute to ecosystem and human health and ultimately planetary health. A recent study highlights the significance of soil biodiversity and soil health in sustaining primary productivity across various land-use types.^160^ Previous research has demonstrated that soil is a significant source of bacteria associated with fruits and vegetables.^28,161,162^ Consequently, maintaining soil health and supporting the soil microbiome will be essential for plant health, as well as for promoting bacterial diversity in our fruits and vegetables, which may also have implications for gut health. This connection highlights the complex interrelationships between our environment, the food we farm, transport, eat, and our overall health (Figure 3).

There is an urgent need to transition toward sustainable agricultural practices that can support and promote biodiversity. This includes increasing crop and cultivar diversity, reducing the use of fertilizers and pesticides, and adopting methods such as crop rotation, agroforestry, intercropping, minimal tillage, and organic farming.^163^ For instance, a recent systematic review indicates that an immediate reduction in pesticide usage presents viable options for enhancing the biodiversity of key aboveground and belowground taxonomic groups, including birds, mammals, arthropods, nematodes, earthworms, and soil microorganisms.^164^ Moreover, organic farming has been proposed as a pivotal strategy to combat or potentially reverse biodiversity loss.^165^ These practices have also been reported to increase the microbial diversity of fruits and vegetables.^23,49,166^ It is also important to educate the public about the importance of microbial diversity in our diet and its implications for health and the environment can encourage healthier food choices and support sustainable practices. Fresh fruits and vegetables are stored and shipped long distances before reaching the consumer point-of-purchase. Extended storage and shipping durations necessitate the use of pesticides for preservation, which can effectively reduce microbial presence but may also unintentionally eliminate beneficial microbiota. We suggest that consuming more plant-based foods and prioritizing fresh, locally sourced options can significantly reduce carbon footprints and mitigate climate impact as well as offer healthier options and beneficial microbiota, ultimately contributing to the health of our planet. Therefore, we recommend integrating the edible plant microbiome within the frameworks of the exposome and One Health concepts, highlighting the interconnections between microbial communities in our food systems, human health, and the environment.

## Challenges and outlook on the future of the edible plant microbiome

The impact of food on gut health is a popular topic in public health. However, some fundamental questions that remain largely unresolved, and some priority areas for future research will be suggested:

1. The scientific evidence supporting the transmission of beneficial bacteria from fruits and vegetables to the human host and their ecological impact on the gastrointestinal system is currently limited. Given that gut microbiome diversity is connected to the development of various chronic conditions, and that microbes derived from fruits and vegetables can enhance gut microbiome diversity, it may be worthwhile to conduct targeted studies to explore the impact of the edible plant microbiome on cohorts with chronic diseases. Overall, further research is necessary to understand their impact on human health, and the most effective approaches to incorporating them into our diets.
2. Besides bacteria as the dominant member, fungi constitute approximately 0.1% of the total gut microbiome.^167^ In healthy adults, in comparison to the bacterial diversity, fungal diversity is low in the lower gastrointestinal tract, with significant variability within and between individuals.^168^ A recent study has reported on the potential acquisition of *Saccharomyces cerevisiae* strains from food sources that have already been reported.^88^ However, the transmission of fungi from fruits and vegetables to the human gut as well as their ecological impact remains largely unexamined.
3. Fruits and vegetables have the potential to serve as an innovative source of probiotics. However, traditional methods for identifying isolates with these characteristics tend to be time-consuming and labor-intensive. To address this, high-throughput screening can be employed to efficiently screen bacterial isolates for probiotic features while reducing costs and labor requirements. Despite its potential, a well-controlled intervention study is needed to demonstrate health benefits from strains derived from fruits and vegetables and their safety.
4. Research on the mechanistic processes and impact of the edible plant microbiome on the human host by using animal models and clinical studies are needed to understand their impact on human health. A crucial question here is whether the microorganisms colonize the intestine transiently or permanently. Therefore, longitudinal study is essential to gather valuable information regarding the temporal trends of colonization and to identify personalized responses related to the edible plant microbiome. By comparing the genetic sequences of the bacteria present in both the food source and the host, we can also identify similarities that indicate a direct link between the two and confirm that the bacteria originated from the consumed food. This can be done by coupling shotgun metagenome data, along with strain-level profiling tools like StrainPhlAn4^169^ and inStrain.^170^ This methodology will facilitate the investigation of the establishment and persistence of plant-associated bacteria sourced from food within the human gut.
5. Understanding the influence of human activities on plant microbiomes is also crucial for maintaining the health and safety of both plants and humans. Attention should be placed on understanding the extent of diversity in the edible plant microbiome and ways to preserve it since recent calls addressing environmental conservation have not considered these activities.
6. The edible plant microbiome can also be further developed as an indicator for food quality and used for monitoring along the food chain. This suggests improving current food safety regulations and extending them by a holistic view of the impact of the microbiome.
7. The edible plant microbiome can be integrated into personalized medicine and diets. While potential beneficial bacteria were already identified in common fruits, the full spectrum of probiotics, bioactive and health-supporting compounds in the edible microbiome is still waiting for discovery. Food labels and daily dietary consumption recommendations will help to recognize its importance and promote its adoption.
8. It is important to acknowledge that while the gut microbiome has been reported to be associated with human health, some meta-analyses and cross-disease comparisons have indicated that many of these associations may be nonspecific or confounded,^171,172^ meaning they could be attributed to other factors that should be considered. Overall, one of the key challenges in microbiome research is distinguishing between correlation and causation within the complex host-microbiome-environment system. To advance the field, it is essential to conduct well-controlled longitudinal cohort studies that monitor gut microbiome changes over time in relation to health outcomes and exposures. Additionally, implementing causal inference methods – such as Mendelian randomization and the use of gnotobiotic animal models – is crucial for validating edible plant microbiome-related effects. Mendelian randomization (MR) methods use genetic variants to evaluate whether an observed association between a risk factor and an outcome suggests a causal relationship.^173^ Moreover, as the edible plant microbiome is a component of our diet, incorporating comprehensive dietary and relevant metadata i.e., raw or processed foods and food origins into analyses can also help account for confounding variables and facilitate the identification of meaningful biological signals. These combined strategies are vital for progressing toward a mechanistic understanding and translating findings into practical applications.
